# Rootstocks Shape Their Microbiome—Bacterial Communities in the Rhizosphere of Different Grapevine Rootstocks

**DOI:** 10.3390/microorganisms9040822

**Published:** 2021-04-13

**Authors:** Leonie Dries, Simone Bussotti, Carlo Pozzi, Robert Kunz, Sylvia Schnell, Otmar Löhnertz, Anne Vortkamp

**Affiliations:** 1 Department of Soil Science and Plant Nutrition, Hochschule Geisenheim University, 65366 Geisenheim, Germany; Simone.bussotti.enol@gmail.com (S.B.); Robert.kunz@hs-gm.de (R.K.); Otmar.loehnertz@hs-gm.de (O.L.); 2 Department of Agricultural and Environmental Sciences, University of Milano, 20133 Milano, Italy; carlo.pozzi@unimi.it; 3 Research Center for BioSystems, Land Use, and Nutrition (IFZ), Institute of Applied Microbiology, Justus-Liebig University Giessen, 35392 Giessen, Germany; sylvia.schnell@umwelt.uni-giessen.de; 4 REACH EUREGIO Start-up Center, University of Muenster, 48151 Muenster, Germany; Anne.Vortkamp@wiwi.uni-muenster.de

**Keywords:** viticulture, metabarcode sequencing, microbiota, soil, vineyard soil

## Abstract

The microbiota associated with the rhizosphere is responsible for crucial processes. Understanding how the plant and its bacterial community interact is of great importance to face the upcoming agricultural and viticultural challenges. The composition of the bacterial communities associated with the rhizosphere of grapevines is the result of the interaction between many drivers: biogeography, edaphic factors, soil management and plant genotype. The experimental design of this study aimed to reduce the variability resulting from all factors except the genotype of the rootstock. This was made possible by investigating four ungrafted grapevine rootstock varieties of the same age, grown on the same soil under the same climatic conditions and managed identically. The bacterial communities associated with the rhizosphere of the rootstocks 1103 Paulsen, 140 Ruggeri, 161-49 Couderc and Kober 5BB were characterized with the amplicon based sequencing technique, targeting regions V4–V5 of 16S rRNA gene. Linear discriminant analysis effect Size (LEfSe) analysis was performed to determine differential abundant taxa. The four rootstocks showed similarities concerning the structure of the bacteria assemblage (richness and evenness). Nonetheless, differences were detected in the composition of the bacterial communities. Indeed, all investigated rootstocks recruited communities with distinguishable traits, thus confirming the role of rootstock genotype as driver of the bacteria composition.

## 1. Introduction

The continuous improvement of cultivation-independent techniques is driving a paradigm shift in the field of biology; the expression of a specific phenotype is no longer determined solely by the interaction between genotype and environment but has to take into account the host-associated microorganisms (microbiota) [1]. The microbiome (the genome of the microbiota) provides adaptability and metabolic diversity, broadening the plant capacity to overcome environmental changes and to cope with challenging conditions [2,3]. The microbes can be divided in epiphytes when they colonize the exterior surfaces or in endophytes when they colonize the interior surfaces [4]. Moreover, the distribution of the microbiota is not even throughout the plant, which can be interpreted as an assembly of several niches. The main distinction is between the above ground and the belowground part. However, agricultural productivity is based on microbial activity, much of which occurs in the soil [5]. The productivity of agricultural systems is highly dependent on the functional processes of microbial communities in the soil, more specifically in the rhizosphere [6]. In particular, the rhizosphere microbiome can positively influence plant development, overall vigor and plant growth by providing nutrients, and has generally been targeted to identify positive relationships between plants and microorganisms [7,8]. However, even if there are no physical barriers, the composition of the soil microbiota is not the same as the rhizosphere microbiota. The soil serves as the initial inoculum, but then the plant actively recruits the bacteria from the soil [9]. Thus, the rhizospheric communities are a subset of the more complex soil microbiota [10]. This selection occurs at the level of the root compartment, which consists of the rhizosphere, rhizoplane and root endosphere [11,12]. Due to this selection, the choice of the right rootstock for recruitment of the soil microbiota is crucial, which is also relevant for grapevine. Even though rootstocks were adopted for their resistance to *Phylloxera*, it is necessary to take into account several other factors, such as grafting compatibility, rooting and propagation ability, resistance to nematodes and pathogens, tolerance to lime, salinity, drought and nutrient uptake [13]. However, the impact of genetic variation on the composition of the microbiome of grapevine species, particularly in the root compartment, is poorly studied [14]. And it can be expected that different rootstocks differentially select their microbial communities from the surrounding soil [4]. Consequently, rootstock genotype affects the selection and recruitment of bacteria that colonize aboveground plant organs such as flowers and fruits and can alter fruit quality [4,15]. Thus, rootstocks could play a role in determining the plant-associated bacterial community by affecting the microbial terroir [16]. This study considered rootstocks derived from the breeding of the most commonly used *Vitis riparia*, *Vitis berlandieri* and *Vitis rupestris* [17]. The experimental design of this study aimed to reduce the variability resulting from environmental factors to unravel the effect of the genotype of the grapevine rootstock on the bacterial communities in the rhizosphere. This was made possible by using ungrafted grapevines of the same age, grown in the same soil under the same climatic conditions and managed identically.

## 2. Materials and Methods

### 2.1. Vineyard Location and Vines

**Figure 1:**
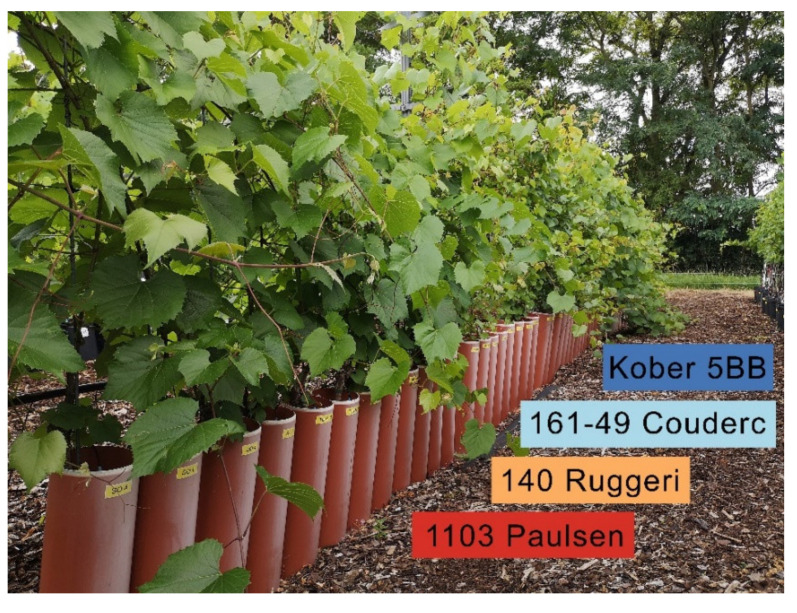
Experimental vineyard. The grapevine rootstocks are planted in cylindrical tubes with a diameter of 15 cm and a height of 120 cm.

The experiment site was located at Geisenheim University, Germany (49°59′N, 7°57′E; 96 m above sea level) in the wine-growing region of Rheingau. The climate of the region is temperate oceanic (Köppen-Geiger classification: Cfb) [18]. The mean annual temperature of the 1986–2010 period is 10.5 °C and the total annual precipitation averages 543.0 mm [19]. The grapevines were planted in a soil mixture (two parts loess soil with one part sand) in cylindrical tubes with a diameter of 15 cm and a height of 120 cm in the year 2015 (Figure 1). The tubes are perforated at the bottom side allowing the water to drain. The following rootstocks were selected for the investigations: 1103 Paulsen (*V. berlandieri* x *V. rupestris*, clone 1 Gm, “1103 P” when abbreviated), 140 Ruggeri (*V. berlandieri* x *V. rupestris* clone 4 Gm, “140 Ru” when abbreviated), 161-49 Couderc (*V. berlandieri* x *V. riparia* clone 3 Gm, “161-49 C” when abbreviated) and Kober 5 BB (*V. berlandieri x V. riparia* clone 13-3 Gm).

### 2.2. Rhizosphere and Soil Sampling

Rhizosphere and soil sampling was performed in July 2020. The tubes with the grapevines were extracted from the ground. Subsequently, each tube was opened using an angle grinder, which completely exposed the root system. This procedure ensured accurate selection and sampling of the rhizosphere soil, which was taken in the very close proximity to the roots. Three replicates per rootstock genotype were investigated. For each replicate, two rhizosphere soil samples (>2 g each) for microbiome analyses were collected and stored at −80 °C. Additionally, for each replicate one soil sample (>100 g) was collected for soil analyses and stored at 4 °C.

### 2.3. Soil Analyses

All soil analyses were performed at the Department of Soil Science and Plant Nutrition, Hochschule Geisenheim University. The soil samples were dried for three days at 30 °C and sieved to a size of 2 mm. For all soil samples (12 samples, n = 3 per rootstock) the pH level as well as the concentration of phosphorus (P), potassium (K) and magnesium (Mg) were measured as described previously by Hendgen et al. [20], according to the VDLUFA database of methods [21]. Statistical analyses were conducted with Microsoft^®^ Excel^®^.

### 2.4. DNA Extraction, Sample Preparation and Ion Torrent Sequencing

DNA from 500 mg rhizosphere soil per sample was extracted according to Lueders et al. [22] and Mettel et al. [23] with the following modification: instead of the described sodium dodecyl sulfate (SDS) solution, TPM (50 mM TrisHCl, 1.7% Polyvinylpyrrolidone, 20 mM MgCl_2_) was used. Extracted DNA was solved in water and stored at −80 °C. The Ion Torrent Polymerase-Chain-Reactions (PCRs) were conducted as described in Kaplan et al. [24]. The first Ion Torrent PCR was performed with KAPA HiFi Polymerase kit (Roche Sequencing Store, Basel, Switzerland) according to the manufacturer’s protocol. During amplification of the partial sequence of the hypervariable regions (V4-V5), the primer 926R (5′-CCGYCAATTYMTTTRAGTTT-3′ [25]) and 520F (5′-AYTGGGYDTAAAGNG-3′ [26]) were used. Amplification parameters were 3 min at 95 °C followed by 35 cycles at 98 °C for 20 s, 55 °C for 30 s, 72 °C for 30 s and finally 72 °C for 5 min. The amplified PCR product was confirmed with agarose gel electrophoresis and used as the template of the second Ion Torrent PCR reaction and barcoding according to Kaplan et al. [24]. The PCR was performed with the following amplification parameters: 3 min at 95 °C followed by 10 cycles at 98 °C for 20 s, 55 °C for 30 s, 72 °C for 30 s and finally 72 °C for 7 min. The PCR products were loaded onto an agarose gel and subsequently purified using NucleoSpin^®^ Gel and PCR Clean-up (MACHEREY-NAGEL GmbH & Co. KG, Düren, Germany). Further, this product was purified with DNA purification beads NucleoMagVR NGS clean-up kit (MACHEREY-NAGEL GmbH & Co. KG, Düren, Germany). The Ion Torrent Sequencing was done according to Kaplan et al. [24].

### 2.5. Bioinformatic Analysis of the Sequencing Data

Bioinformatic analysis was performed with QIIME 2 2020.8 [27]. The obtained raw sequences were demultiplexed using cutadapt [28] with no errors allowed in the barcode sequences. Quality control, sequence denoising, clustering to amplicon sequence variants (ASVs) (i.e., 100% operational taxonomic units) [29], dereplication and removal of chimera sequences were done with DADA2 [30] (via q2-dada2). Thereby the first 15 bp were deleted and all sequences were cut to a length of 330 bp. Taxonomy was assigned to the ASVs using the q2-feature classifier [31], which uses naïve Bayes machine-learning classifiers [32] to assign taxonomies based on sequence k-mer frequencies. As a reference database, a trained version of the Silva ribosomal database [33] version 137 was used. All ASVs belonging to chloroplasts and mitochondria were removed. All ASVs were aligned with mafft [34] (via q2-alignment) and used to construct a phylogeny with fasttree2 [35] (via q2-phylogeny). Alpha-diversity metrics (Faith’s Phylogenetic Diversity [36]—measures of microbiome richness, and Pielou’s Evenness) were calculated in R version 4.0.2 [37] using the packages phyloseq version 1.34.0 [38] and picante version 1.8.2 [39]. Beta-diversity metrics (weighted UniFrac [40], unweighted UniFrac [41] and Bray-Curtis dissimilarity [42]—measures of microbiome composition dissimilarity), and Principal Coordinate Analysis (PCoA) were estimated using q2-diversity after samples were rarefied to 2220 sequences per sample. The distance matrices were also analyzed by the PERMANOVA test [43], followed by the PERMDISP test [44] to ensure the homogeneity of dispersion among the four rootstocks. Both the PERMANOVA and PERMDISP tests were performed using 999 permutations. Linear discriminant analysis effect Size (LEfSe) was run to assess differences in bacteria distribution among the rootstocks [45]. The online Galaxy platform [46] was used. The threshold for the logarithmic linear discriminant analysis (LDA) score was set at 2.0 and the non-parametric factorial Kruskal Wallis sum-rank test (α = 0.05) was performed with an all-against-all strategy. Significant taxa were visualized with a bar graph and in a taxonomic cladogram.

## 3. Results

### 3.1. Soil Analyses

The main chemical characteristics of the sampled soils were measured and a Kruskal-Wallis H test was conducted. The pH levels (Table 1) ranged between 7.53 ± 0.09 and 7.60 ± 0.00 without a statistical difference (*p* = 0.89). The P, K and Mg contents (Table 1) were also statistically similar (respectively, *p* = 0.34, p = 0.63, *p* = 0.15).

### 3.2. High-Throughput Amplicon Sequencing

The Ion Torrent sequencing generated 910,225 16S rRNA gene V4-V5 amplicon sequences, 72% of which (642,114) passed the Ion Torrent Sequencing quality selection. After barcoding errors cleaning, the remaining 578,539 reads were demultiplexed to assign each read to the respective sample. Four samples were removed from the subsequent analysis due to the low number of reads (<2827 features; one sample per rootstock). The denoised and filtered reads were clustered into 2034 amplicon sequence variants (ASVs).

### 3.3. Diversity and Richness of Bacterial Communities

**Figure 2:**
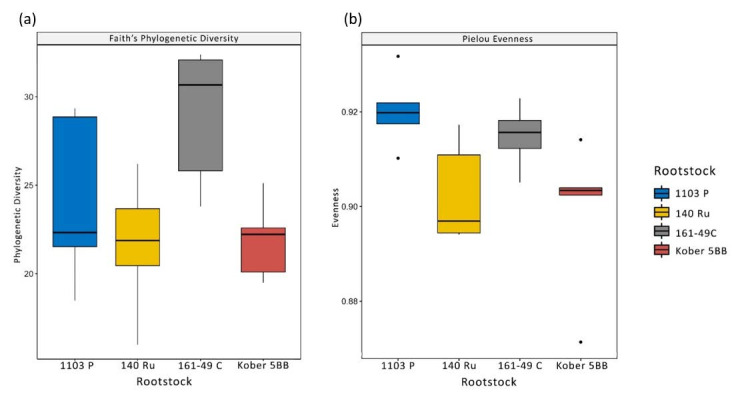
Boxplots reporting the results of alpha diversity analysis. (**a**) Faith’s phylogenetic plot showing similar values for the four rootstocks. (**b**) Pielou’s evenness plot showing values higher than 0.895 for all the rootstocks.

**Figure 3:**
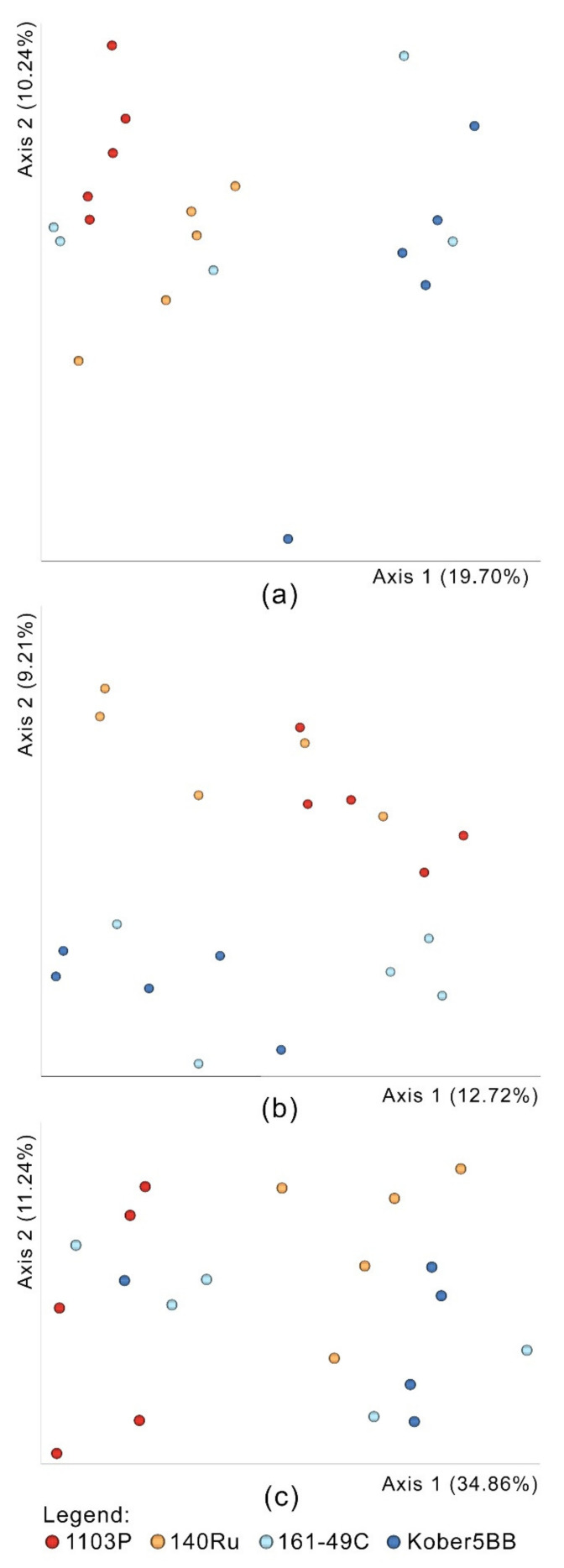
Principal Coordinate Analyses (PCoAs) reporting the distance matrices of the beta diversity indexes: (**a**) Bray-Curtis, (**b**) unweighted UniFrac and (**c**) weighted UniFrac.

Pielou’s evenness and Faith’s phylogenetic diversity were used to calculate alpha-diversity (i.e., the diversity within the group). All measures indicated that no significant differences between the structure of the rhizosphere communities were observable (Figure 2). Pielou’s evenness reported values approaching 1, meaning that bacteria were evenly distributed among species. To calculate beta-diversity (i.e., the diversity between groups) the estimators Bray-Curtis, unweighted UniFrac and weighted UniFrac were used. The distance matrices were ordered on a PCoA (Figure 3). The permutation test with pseudo-F ratios revealed a significant effect of the factor “rootstock genotype” on beta-diversity for Bray-Curtis (*p* = 0.001), unweighted UniFrac (*p* = 0.001) and weighted UniFrac (*p* = 0.002) indices. PERMDISP was run to assess the homogeneity of the dispersion. The test led to a p-value of 0.131 for Bray-Curtis, 0.572 for unweighted UniFrac and 0.258 for weighted UniFrac.

### 3.4. Taxonomy and Distribution among Rootstocks

**Figure 4:**
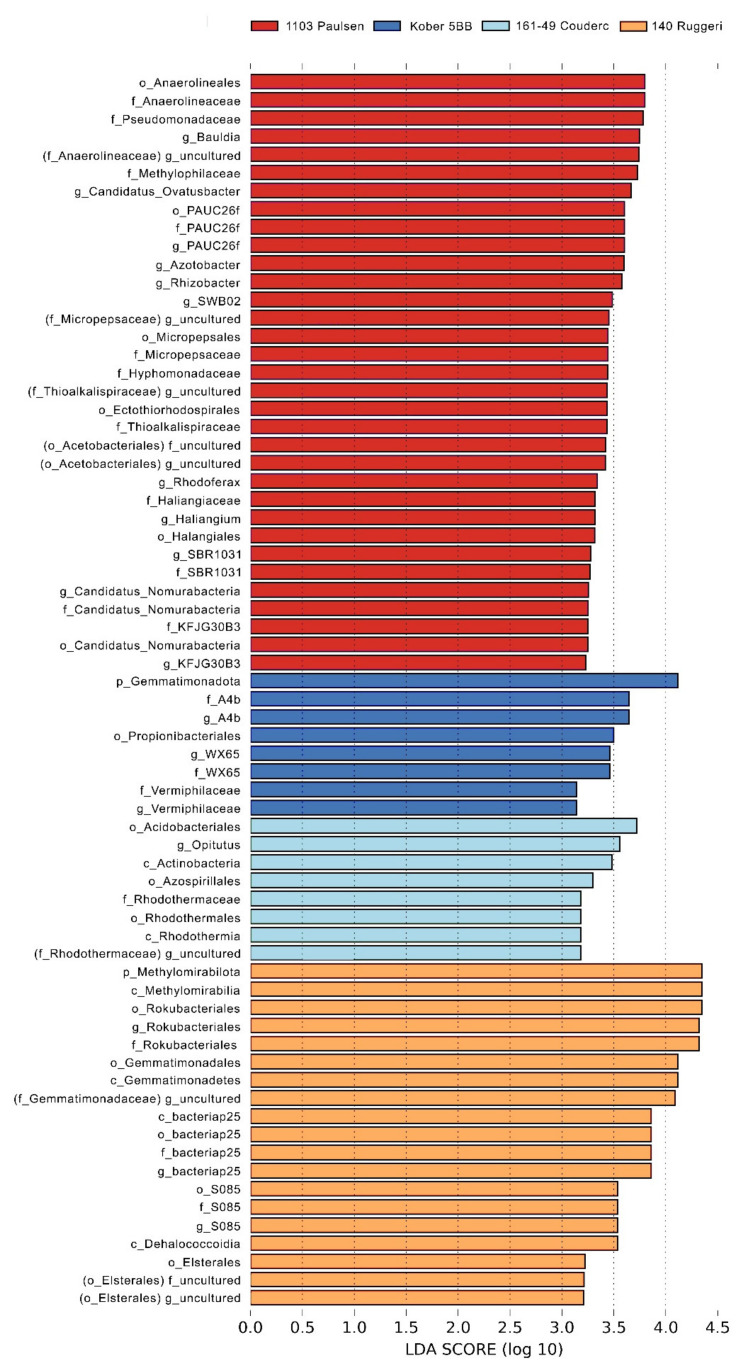
Histogram of the linear discriminant analysis (LDA) scores reveals the most differentially abundant taxa among the four rootstocks 1103 Paulsen, 140 Ruggeri, 161-49 Couderc and Kober 5BB.

**Figure 5:**
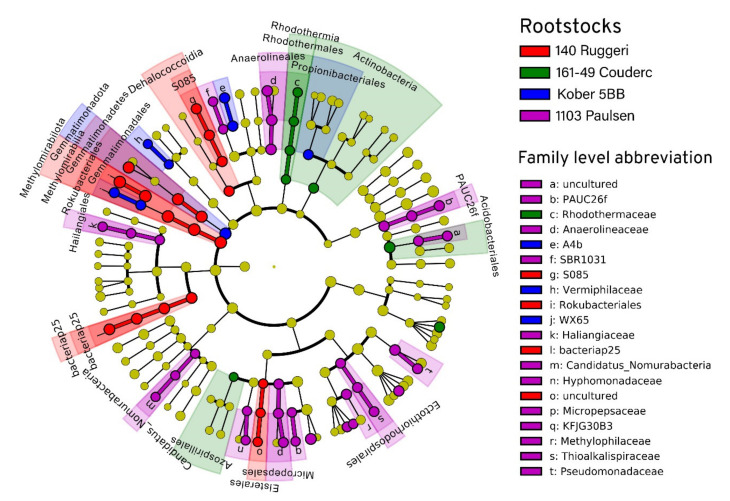
Linear discriminant analysis (LDA) effect size taxonomic cladogram comparing the bacterial diversity of the four rootstocks. Each ring represents a taxonomic level, with kingdom, phylum, class, order, family and genus appearing from the center to the periphery. Each node represents a taxonomic unit found in the dataset. For each significantly discriminating taxon detected, the corresponding node and branch region in the taxonomic cladogram is colored according to the highest ranked group for that taxon. If the taxon is not significantly different between rootstocks, the corresponding node is colored yellow.

The taxonomy was assigned using a trained classifier against the Silva database. The 2034 different ASVs, all belonging to the bacteria domain, clustered into 36 different phyla, 97 classes, 199 orders, 257 families and 365 genera (Supplementary Materials Table S1). The rhizosphere was dominated by *Acidobacteriota* (35%), *Proteobacteria* (22%), *Latescibacteriota* (15%), *Methylomirabilota* (6%) and *Gemmatimonadota* (4%). The most represented classes were *Vicinamibacteria* (17%), *Gammaproteobacteria* (15%), *Latescibacterota* (15%), *Acidobacteriae* (9%), *Alphaproteobacteria* (7%), *Methylomirabilia* (6%) and *Gemmatimonadetes* (4%). The linear discriminant analysis effect size (LEfSe) detected 68 bacterial clades differently distributed among the rootstocks (Figure 4 and Figure 5). Significant differences (LDA score > 2.0) were found for each taxonomic level: phylum (2), class (6), order (15), family (20) and genus (25). *Proteobacteria* was the most heterogeneously distributed phylum (21 clades, 12 belonging to *Alphaproteobacteria* and 9 belonging to *Gammaproteobacteria*), followed by the phyla *Chloroflexi* (11 clades), *Methylomirabilota* (7 clades) and *Myxococcota* (7 clades). The rootstock 1103 Paulsen reported the highest number of differentially abundant taxa (32).

## 4. Discussion

The experimental design of this study aimed at minimizing the variability from all factors but the rootstock genotype. This was made possible by using grapevines of the same age, located in the same soil sharing also the same climatic conditions, and managed identically. The amplicon sequencing analysis revealed that rootstock rhizospheres recruited complex bacterial communities mainly composed of *Acidobacteriota*, *Proteobacteria*, *Latescibacterota*, *Methylomirabilota* and *Gemmatimonadota*. These results are in partial agreement with the data of other recent studies [4,14,16]. According to Berlanas et al. [14], the dominant phyla found in two vineyards located in Northeastern Spain were *Proteobacteria*, *Actinobacteria*, *Acidobacteria* and *Bacteroidetes*. Similarly, Marasco et al. [16] reported phylum *Proteobacteria* as prevalent, followed by *Actinobacteria*, *Bacteroidetes*, *Chloroflexi* and *Acidobacteria* in an Oltrepò Pavese (Italy) vineyard. These results highlight that there is a core of bacteria always recruited by grapevine despite the different conditions (e.g., biogeography, and climatic or edaphic factors). Among them, *Proteobacteria* is commonly the dominant phylum followed by *Actinobacteria*. Both phyla play an important role in carbon cycling and production of secondary metabolites [47]. *Proteobacteria* comprise organisms with a broad variety of metabolic capabilities; alpha-, beta-, gamma- and delta- *Proteobacteria* are the classes mostly described in soil studies. In this study, only *Gammaproteobacteria* and *Alphaproteobacteria* were found. Both classes are considered copiotrophs (r-strategist) and can easily colonize environments characterized by abundance of nutrients, such as the rhizosphere [48]. Instead, *Actinobacteria* are described as oligotrophic k-strategists growing slowly and with low nutritional requirements [49]. In this project, the percentage of ASVs assigned to this phylum was rather low (0.56%) in comparison to other studies. Conversely, the percentage shown by *Acidobacteriota* phylum was unusually high (~ 35%), and greater than *Proteobacteria* phylum (~22 %). *Acidobacteriota* phylum is one of the most widespread and abundant on the planet [50], but its abundance is reported to decrease moving from bulk soil to rhizosphere [2]. Information about *Acidobacteriota* functionality is scarce since there are issues in cultivating the majority of the members of this phylum [51]. A negative correlation between the abundance of *Acidobacteriota* and concentration of the organic carbon in soil was reported [50]. This, together with the low growth rate, may indicate this phylum as oligotrophic [50]. Additionally, Zarraonaindia et al. [4] reported high abundance of *Acidobacteriota* together with unusually low abundance of *Actinobacteria*. It may be suggested that *Acidobacteriota* can replace *Actinobacteria* when some biotic or abiotic factors hamper the normal thriving of the latter. Indeed, both phyla are described as oligotrophic k-strategist [50] and occupy the same niche. Remarkably, this study is the first to report the occurrence of the phylum *Latescibacterota* (previously known as WS3) among the most abundant phyla in vineyard soils. *Gemmatimonadota* phylum was first associated with vineyard soils by Novello et al. [52], and this study confirmed their presence in the grapevine rhizosphere. *Gemmatimonadota* is reported as one of the top nine phyla found in soils [53]. Nonetheless, little is known about its metabolic capabilities since most of its members are uncultivated. It was observed that the microbial communities associated with the four rootstocks did not significantly differ in evenness and richness. Indeed, all the four rootstocks showed a bacterial community with no clearly dominating species and with similar phylogenetic richness. This is consistent with the theory of a well conserved core of bacteria associated with the grapevine rhizosphere [14]. This study, however, showed that rootstocks 1103P, 140 Ru, 161-49C and Kober 5BB were able to assemble distinct bacterial microbiota at the rhizosphere level. The percentage explained by weighted (46%) and unweighted UniFrac (22%) suggests that the differences are mainly driven by a different recruitment of the same bacteria. Moreover, the LEfSe analysis detected 68 clades significantly differentially recruited by the four investigated rootstocks. These differences confirm the rootstock genotype as a substantial driver of the bacterial communities associated with the rhizosphere. The rootstock genotype may exert its influence on the rhizosphere associated communities through specific root exudates [54,55,56] and by means of its immune system. Indeed, it has been proven that the immune system can affect not only the microbial composition of the root interior surfaces but also the communities thriving near the root [57,58]. Marasco et al. [16] have investigated whether different rootstocks affect the recruitment of the bacteria from the surrounding soil comparing ungrafted and grafted grapevines of the *Vitis vinifera* cultivar Barbera, all cultivated in the same soil. They concluded that the rootstock can influence the diversity and richness of the bacterial communities associated with the root and that the rootstock is a factor determining the specificity of the microbiota independently of the scion cultivar. D’Amico et al. [59] have highlighted the importance of the microbiota recruited by the rootstock with a cultivation-independent technique: the same scion (*V. vinifera* cv Lambrusco) was grafted onto two different rootstocks (1103 Paulsen and Kober 5BB) in the same vineyard, where only the grapevines grafted on 1103 Paulsen had potassium absorption problems. The analysis of the microbiota with an amplicon-based approach has revealed that the rootstock 1103 Paulsen is not able to successfully recruit several potassium solubilizing microorganisms [59]. Thus, the knowledge of the microbiota associated with diverse rootstocks is valuable to make the right choice at the moment of the implant, to intervene when the choice is already taken, and also for breeders to select for rootstocks with a specific microbiota.

## 5. Conclusions

These results show that the rootstock genotype affects the composition but not the structure of the bacterial community in the rhizosphere. However, recognizing the associations between bacteria and rootstock is crucial but still not sufficient to exploit this information from a practical perspective. In conclusion, it is essential to understand the recruitment mechanisms of grapevine to microbial communities and to identify the beneficial taxa in a more detailed context. Following studies should use a whole genome sequencing approach and combine it with other meta -omics methods. For example, the use of metatranscriptomics and metaproteomics together allows the characterization of the microbial communities thriving in the rhizosphere and simultaneously the comprehension of their role. However, applying several -omics methods will inevitably lead to a better comprehension of the rhizosphere microbiome in relation to soil type, grapevine variety, rootstock and environmental conditions.

## Figures and Tables

**Table 1:** Main chemical characteristics of the soil of the four different rootstocks. Phosphorus (P), potassium (K) and magnesium (Mg) values are expressed in mg kg^-1^.

Soil Characteristics	1103 P	140 Ru	161–49C	Kober 5BB
pH	7.53 ± 0.12	7.53 ± 0.09	7.60 ± 0.00	7.57 ± 0.12
P	117.72 ± 9.42	194.75 ± 76.05	152.60 ± 19.82	140.97 ± 52.48
K	165.98 ± 20.33	237.90 ± 137.09	193.64 ± 19.56	157.68 ± 58.68
Mg	116.61 ± 12.39	86.45 ± 52.66	152.79 ± 15.04	138.72 ± 22.57

## Data Availability

The sequencing reads from this study are openly available in the National Center for Biotechnology Information (ncbi) under accession no. PRJNA716534. This data can be found at https://www.ncbi.nlm.nih.gov/bioproject/716534.

## Supplementary Materials

The following are available online at https://www.mdpi.com/article/10.3390/microorganisms9040822/s1, Table S1: Amplicon Sequenced Variants Table.
